# The effect of GLP-1 receptor agonist lixisenatide on experimental diabetic retinopathy

**DOI:** 10.1007/s00592-023-02135-7

**Published:** 2023-07-09

**Authors:** Kuebra Oezer, Matthias Kolibabka, Johann Gassenhuber, Nadine Dietrich, Thomas Fleming, Andrea Schlotterer, Michael Morcos, Paulus Wohlfart, Hans-Peter Hammes

**Affiliations:** 1 5th Medical Department, Medical Faculty Mannheim, Heidelberg University, Mannheim, Germany; 2 Sanofi, MSAT M&I Bioassays and Compliance, Frankfurt, Germany; 3 Department of Medicine I and Clinical Chemistry, Heidelberg University, Heidelberg, Germany; 4 German Center for Diabetes Research (DZD), Neuherberg, Germany; 5 Stoffwechselzentrum Rhein-Pfalz, Belchenstraße 1-5, 68163 Mannheim, Germany

**Keywords:** Diabetic retinopathy, Neurovascular unit, GLP-1RA, Lixisenatide, ROS, Ets2

## Abstract

**Aims:**

Glucagon-like peptide-1 receptor agonists are effective treatments for type 2 diabetes, effectively lowering glucose without weight gain and with low risk for hypoglycemia. However, their influence on the retinal neurovascular unit remains unclear. In this study, we analyzed the effects of the GLP-1 RA lixisenatide on diabetic retinopathy.

**Methods:**

Vasculo- and neuroprotective effects were assessed in experimental diabetic retinopathy and high glucose-cultivated C. elegans, respectively. In STZ-diabetic Wistar rats, acellular capillaries and pericytes (quantitative retinal morphometry), neuroretinal function (mfERG), macroglia (GFAP western blot) and microglia (immunohistochemistry) quantification, methylglyoxal (LC–MS/MS) and retinal gene expressions (RNA-sequencing) were determined. The antioxidant properties of lixisenatide were tested in C. elegans.

**Results:**

Lixisenatide had no effect on glucose metabolism. Lixisenatide preserved the retinal vasculature and neuroretinal function. The macro- and microglial activation was mitigated. Lixisenatide normalized some gene expression changes in diabetic animals to control levels. Ets2 was identified as a regulator of inflammatory genes. In C. elegans, lixisenatide showed the antioxidative property.

**Conclusions:**

Our data suggest that lixisenatide has a protective effect on the diabetic retina, most likely due to a combination of neuroprotective, anti-inflammatory and antioxidative effects of lixisenatide on the neurovascular unit.

## Introduction

Diabetes mellitus (DM) is characterized by hyperglycemia which causes serious damages to many of the body´s system and causes macrovascular complications (e.g., strokes, myocardial infarctions) and microvascular complications such as neuropathy, nephropathy and retinopathy [1]. Hyperglycemia induces mitochondrial overproduction of reactive oxygen species (ROS) and subsequent formation of methylglyoxal (MG) as well as accumulation of MG-derived advanced glycation endproducts (AGEs). This can lead to endothelial dysfunction, which contributes to the development of diabetic retinopathy [2, 3]. Diabetic retinopathy (DR) is a disease of the neurovascular unit (NVU) with the pathogenic hallmarks of glial activation, vasoregression and neurodegeneration, the latter preceded or accompanied by neuronal dysfunction [3].

Glucagon-like peptide 1 (GLP-1) improves glycemic control in type 2 diabetes (T2D). It has an effect on glucose-stimulated insulin secretion, gastric emptying and hepatic glucose production [4]. Additionally, GLP-1 can block the overproduction of ROS and downstream expression of pro-inflammatory effectors by endothelial cells [5].

GLP-1 demonstrates antioxidative effects on the vasculature and leads to increased cell survival and reduced apoptosis in animal models of diabetes [6, 7]. It was also shown that intravitreal injection of GLP-1 transiently improves neuronal function and reduces glutamate toxicity in the retinae of diabetic mice [8].

In clinical and preclinical studies, GLP-1 treatment reduced inflammatory signaling in macrophages, reduced blood pressure and improved plasma lipid profiles [9–11].

There are drugs that mimic the action of GLP-1 (glucagon-like peptide-1) in the body. For example, the GLP-1 receptor agonist lixisenatide. The anti-diabetic drug activates the GLP-1 receptor and leads to increased insulin secretion, reduces glucagon secretion and slows down gastric emptying. This leads to improved blood sugar control in type 2 diabetes [12–15]. Apart from its effects in T2D patients, GLP-1 receptor agonists are also beneficial in several neurodegenerative and neuroinflammatory diseases [16].

Our previous study showed that chronic treatment of diabetic rats with DPP-IV inhibitor linagliptin led to a marked decrease in the number of retinal acellular capillaries and a significant drop in elevated methylglyoxal levels [17]. In addition, intravitreal injection of exenatide (GLP-1 RA) was shown to protect neuronal function in short term [18].

Given the role of the neurovascular communication in diabetic retinopathy, and the potential to interfere with important pathogenetic pathways in DR, in particular oxidative stress, it is conceivable that lixisenatide protects from diabetes-induced retinal damage.

The aim of the study was to assess lixisenatide on the neurovascular unit of diabetic retinopathy in the STZ-diabetic rat. The second model was *C. elegans* as a surrogate for glucose-induced neurodegeneration. The lack of GLP-1R orthologue in *C. elegans* allows the study of GLP-1 RAs, such as lixisenatide, to be separated from its GLP-1R-mediated effects.

## Materials and methods

### Wistar-rats and experimental groups

Eight-weeks-old male Wistar rats (Harlan Winkelmann, Borchen, Germany) were rendered diabetic by intravenous injection of streptozotocin (STZ) (35 mg/kg body weight; Roche Diagnostics GmbH, Mannheim, Germany). Animals were held in a 12-h light–dark cycle with food and water ad libitum. Rats with a blood glucose level above 14 mmol/L (250 mg/dL) 1 week after STZ injection were considered diabetic (DC). Lixisenatide (Sanofi, Frankfurt) was administered 5 days a week to diabetic rats subcutaneously at a concentration of 150 µg/kg body weight from week 1 to week 12 (DC + Lixi) after STZ treatment. Hemoglobin A1c (HbA1c) was determined using an A1cNow + System from pts Diagnostics (EuroMedix Health am Dom GmbH, Köln, Germany). Age-matched male Wistar rats served as controls (NC). After a diabetes duration of 12 weeks, rats were killed, and the eyes were obtained and stored at − 80 °C until further use.

The study was approved by the regional ethics committee (Regierungspräsidium Karlsruhe, Germany; protocol code “Aktenzeichen: 35-9185.81/G-15/15”; date of approval 16.07.2015). All experiments were conducted in accordance with the Association for Research in Vision and Ophthalmology statement for the Use of Animals in Ophthalmic and Vision Research.

### Retinal digest preparation and quantitative retinal morphometry

For quantification of the retinal vasoregression, we used quantitative retina morphometry as previously described [19]. For this purpose, the rat retinae were isolated, digested, PAS Haemalaun-stained and subsequently evaluated.

### Multifocal electroretinography (mfERG) and optical coherence tomography (OCT)

In-vivo analyses were conducted using the RETImap system (Roland Consult, Brandenburg a.d. Havel, Germany). Neuro-retinal function was quantified with a 7-segment mfERG as previously described [20]. Retinal thickness was measured with the OCT module of the system at the border of the inner third and outer two thirds of the retina.

### GFAP western blot

To analyze retinal Müller glia activation, for western blots the isolated retinae were homogenized in 90 μl 0.1% SDS lysis buffer and protein concentration was determined by Direct Detect Spectrometer (Merck KGaA, Darmstadt, Germany). Samples were separated in a 4–20% TGX Gel (Bio-Rad Laboratories, München, Germany) and immunoblotted with Bio-Rad Trans-Blot Turbo to a polyvinylidene difluoride membrane (Bio-Rad Laboratories, München, Germany). Non-specific binding was blocked by incubation with 5% non-fat dry milk in TBS, containing 0.1% Tween (Sigma-Aldrich, Darmstadt, Germany), followed by overnight incubation at 4 °C with rabbit anti-rat glial fibrillary acidic protein (GFAP) (1:400, Santa Cruz, Heidelberg, Germany) or rabbit anti-rat beta Tubulin (1:500, Abcam, Cambridge, UK) antibodies.

For detection, an anti-rabbit IgG HRP antibody (1:3000, Dako Cytomation, Hamburg, Germany) for GFAP and for beta Tubulin were used. Immunoreactive bands were visualized by incubation with chemiluminescence reagent (Perkin Elmer, Boston, MA, USA) and signals were detected with the Fusion SL (VWR, Darmstadt, Germany). Integrated densities were measured with ImageJ software [21].

### Immunofluorescence for microglial activation

To evaluate retinal microglial activation, retinal whole-mount stainings with CD74, Iba1 and Lectin were performed. Eyes were fixed overnight in 4% formalin at 4 °C. After dissection, retinae were washed in PBS and incubated in the permeabilization and blocking buffer containing 1% BSA and 0.5% Triton-X100 in PBS for 1 h at room temperature. Retinae were incubated at 4 °C overnight with the following primary antibodies CD74 (1:100, Santa Cruz, California), Iba1 (1:100, WAKO Chemicals, Japan) and Lection-biotin (1:50, Sigma, Germany). After washing in PBS, the retinae were then incubated in the following secondary antibodies: Alexa Fluor 488 chicken anti-goat (1:200, Thermo Fisher, Germany) for CD74, Alexa Fluor 555 donkey anti-rabbit (1:100, Thermo Fisher, Germany) for Iba1 and Alexa Fluor 633 StreptAvidin (1:500, Thermo Fisher, Germany) for Lectin at room temperature for 1 h. The retinae were washed in PBS and then flat-mounted in 50% glycerol. Photographs were taken using a confocal microscope (Leica TCSP8, Wetzlar, Germany) and microglial cells positive for CD74 + and Iba1 were quantified per mm^2^ in whole retinae.

### Determination of methylglyoxal levels

Methylglyoxal in retina tissue was determined by isotope dilution, tandem mass spectroscopy, following derivatization with 1,2-diaminobenzene [22]. Briefly, pre-weighted amounts of tissue (ca.10 mg) were homogenized in ice-cold 20% (wt/vol) trichloroacetic acid in 0.9% (wt/vol) sodium chloride (20 µl) and water (80 µl). An aliquot (5 µl) of the internal standard (13C 3 -MG; 400 nM) was then added and the samples vortexed mixed. Following centrifugation (14,000 rpm; 5 min @ 4 °C), 35 µl of the supernatant was transferred to an HPLC vials containing a 200 µl glass interest. An aliquot (5 µl) of 3% sodium azide (wt/vol) was then added to each sample followed by 10 µl of 0.5 mM DB in 200 mM HCl containing 0.5 mM diethylenetriaminepentaacetic acid (DETAPAC) in water. The samples were then incubated for 4 h at room temperature, protected from the light. Samples were then analyzed by LC–MS/MS using an ACQUITY™ ultra-high-performance liquid chromatography system with a Xevo-TQS LC–MS/MS mass spectrometer (Waters, Manchester, UK). The column was a Waters BEH C18 (100 × 2.1 mm) and guard column (5 × 2.1 mm). The mobile phase was 0.1% formic acid in water with a linear gradient of 0–100% 0.1% formic acid in 50% acetonitrile:water over 0–10 min; the flow rate was 0.2 ml/min and column temperature was 5 °C. The capillary voltage was 0.5 kV, the cone voltage 20 V, the interscan delay time 100 ms, the source and desolvation gas temperatures 150 and 350 °C, respectively, and the cone gas and desolvation gas flows were 150 and 800 l/h, respectively. Mass transitions (parent ion > fragment ion; collision energy), retention time, limit of detection, and recoveries were as follows: 145.0 > 77.1, 24 eV, 6.07 min, 0.52 pmol and 98%. Acquisition and quantification were completed with MassLynx 4.1 and TargetLynx 2.7 (Waters^®^).

### RNA-sequencing

Total retinal RNA was isolated using Trizol reagent according to the manufacturer’s protocol (Invitrogen, Carlsbad, CA, USA). Six retinae total RNA samples from each group were selected for RNA-sequencing to obtain an unbiased view of retinal transcriptome. Libraries of mRNA were prepared in a contract-research lab (Atlas Biolabs GmbH, Berlin, 145 Germany) by an RNA sample preparation kit (Illumina, San Diego, CA, USA), and single- 146 end sequenced with a 50 bp fragment size and 40–50 million reads per sample.

### RNAseq data analysis

RNA sequencing raw data were analyzed using a software package and standardized RNA Seq analysis workflow (Array Studio Version 10.1.3.3, Omicsoft Qiagen). This work-flow included alignment to a reference rat gene model, quantification, normalization, and finally detection of differentially expressed genes using the DESeq2 module (EMBL) [23]. Further pathway and in silico analyses were performed using the online software tool GNCPro (SABiosciences) and Astrocyte RNAseq Database.

### *C. elegans* maintenance and experimental groups

The wild-type strain (N2 Bristol) was used for the experiments and was provided by the Caenorhabditis Genetics Center which is funded by National Institutes of Health Office of Research Infrastructure Programs (P40 OD010440). *C. elegans* were cultivated on nematode growth medium (NGM) agar on 60-mm Petri dishes with Escherichia coli bacteria (OP50) as food source and maintained at 20 °C. Self-fertilizing hermaphrodites were removed after allowed to lay eggs to obtain age-synchronized nematodes. After hatching and reaching adulthood, the nematodes were maintained on NGM agar plates containing 300 μg/ml 5-fluorodesoxyuridine (FUdR, Sigma-Aldrich, München, Germany) to prevent hatching. These conditions were used as standard (S). High glucose conditions (HG) were established by transferring adult *C. elegans* to NGM-FUdR plates containing 200 mM glucose. For additional treatment, lixisenatide was added at concentration of 6 µM, 2 µM, 0.6 µM, 0.2 µM and 0.02 µM to the high glucose condition (HG + Lixi). Quantification of ROS formation was performed upon cultivation of animals for 12 days under S, HG or HG + Lixi conditions.

### Quantification of ROS formation

Confocal laser scanning microscopy (CLSM) was used for quantification of ROS formation as described previously [24]. The formation of ROS was detected by fluorogenic oxidation of the O2− sensitive dye hydroethidine to ethidium, according to standard methods. *C. elegans* were washed with phosphate-buffered saline three times and incubated with 3 µM of dihydroethidium for 30 min. We detected ethidium labelled ROS formation (excitation/emission maximum = 518/605 nm) using a 514 nm laser/filter combination and a detector slit of 600–680 nm. Image processing and analysis was performed by using ImageJ software [21]. Background was subtracted from imported raw data and formation of ROS was quantified by calculation of mean pixel values of areas containing single animals.

### Statistical analysis

GraphPad Prism 7.0 software (GraphPad Software Inc., San Diego, California, USA) was used for statistical analysis. Results are reported as means with associated standard deviation (MW ± SD). For comparison from two groups, ANOVA—analysis of variance with Tukey's posttest was performed. A *p* value < 0.05 was considered statistically significant.

## Results

### Metabolic data: lixisenatide treatment has no effect on glucose metabolism

**Fig. 1:**
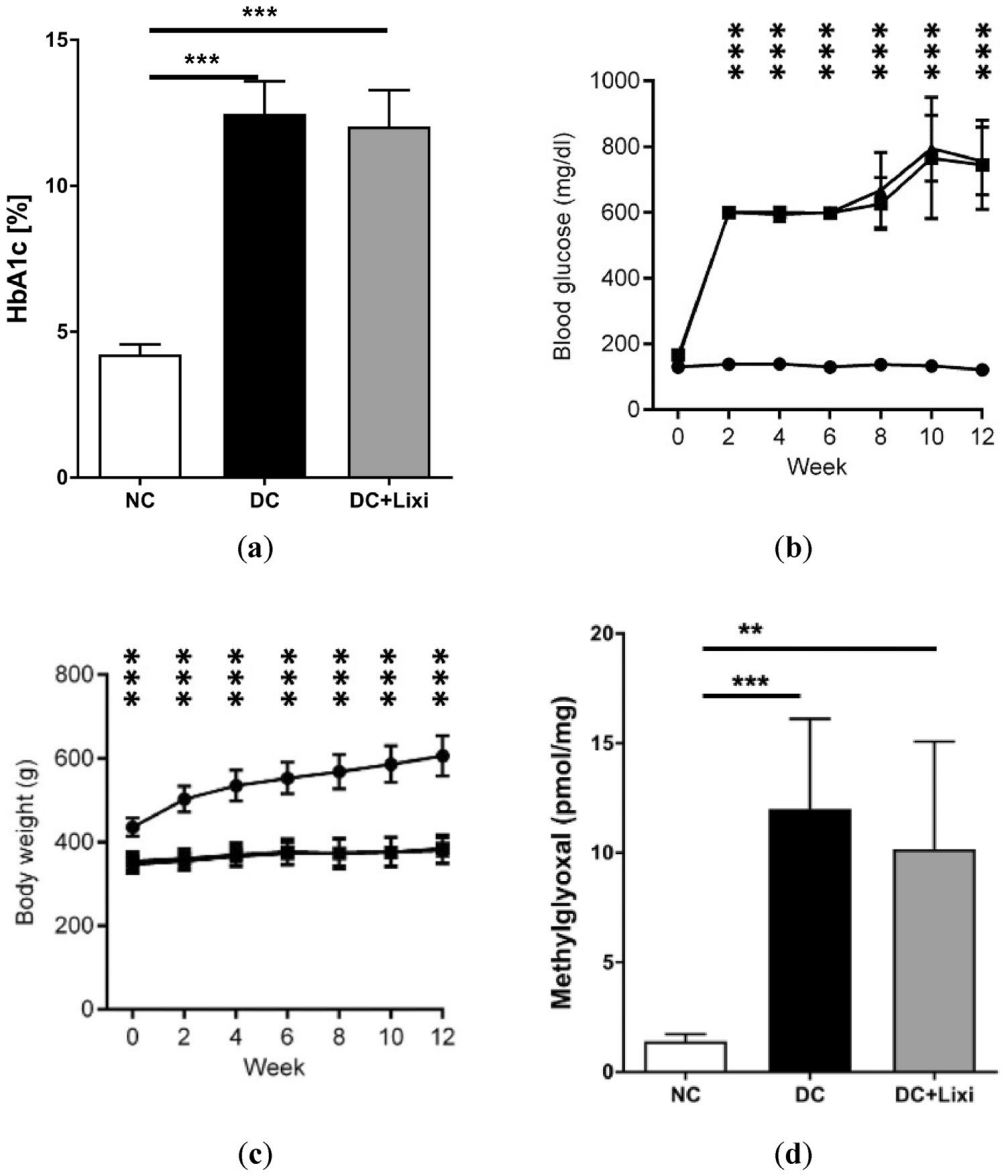
Metabolic and biochemical effects of lixisenatide in diabetic rats. **a** HbA1c (*n* = 6–13, 1way ANOVA, Tukey´s multiple comparisons test, ***p < 0.001), **b** blood glucose (● NC = non-diabetic; ■ DC = diabetic; ▲ DC + Lixi = Lixisenatide-treated diabetic, *n* = 7, 2way ANOVA, Tukey´s multiple comparisons test, ***p < 0.001: significance NC vs. DC and NC vs. DC + Lixi), **c** body weight (● NC = non-diabetic; ■ DC = diabetic; ▲ DC + Lixi = Lixisenatide-treated diabetic, *n* = 9–13, 2way ANOVA, Tukey´s multiple comparisons test, ***p < 0.001: significance NC vs. DC and NC vs. DC + Lixi) and **d** retinal methylglyoxal concentrations (*n* = 6, 1way ANOVA, Tukey´s multiple comparisons test, ***p < 0.001). All data are expressed as mean ± SD

To investigate the metabolic effects of lixisenatide, we analyzed the HbA1c levels, the blood glucose levels, the body weight and retinal methylglyoxal levels. Lixisenatide-treated animals demonstrated no differences in HbA1c levels (NC 4.22 ± 0.35%, DC 12.47 ± 1.11%, DC + Lixi 12.03 ± 1.25%) and blood glucose levels (NC 132.9 ± 6.36 mg/dl, DC 585.9 ± 198.1 mg/dl, DC + Lixi 595.5 ± 209.9 mg/dl, *n* = 7) nor in body weight (NC 540.6 ± 57.32 g, DC 370.6 ± 10.15 g, DC + Lixi 367.0 ± 12.37 g). Of note, retinal methylglyoxal levels were unaffected by lixisenatide treatment in diabetic rats (NC 1.399 ± 0.3376 pmol/mg, DC 11.99 ± 4.119 pmol/mg, DC + Lixi 10.16 ± 4.92 pmol/mg) (Fig. 1a–d).

### Lixisenatide preserves the retinal vasculature and neuroretinal function

**Fig. 2:**
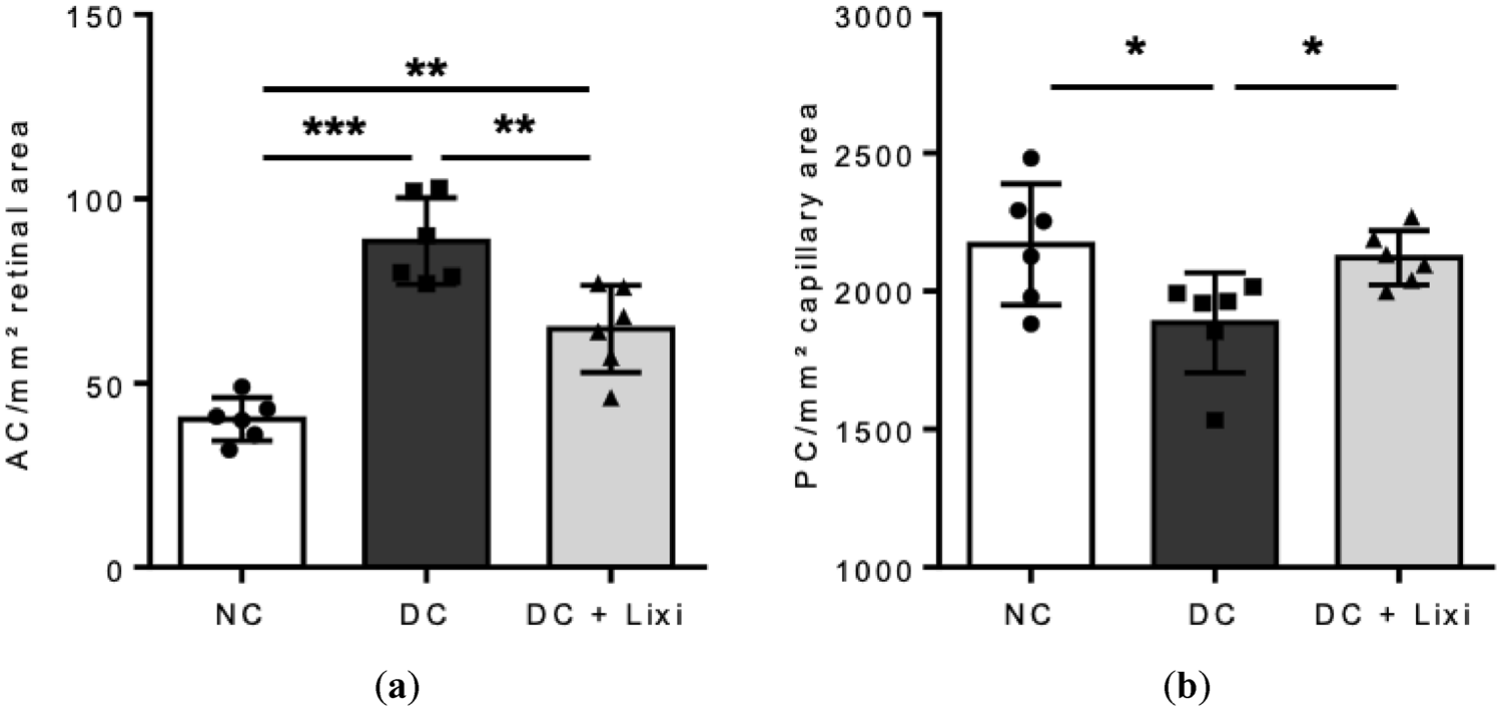
Effect of lixisenatide on experimental diabetic retinopathy. **a** Numbers of acellular capillaries and **b** pericyte numbers in capillary areas were determined by quantitative retinal morphometry of PAS-stained retinal digest preparations. NC = non-diabetic; DC = diabetic; DC + Lixi = Lixisenatide-treated diabetic. Data are expressed as mean ± SD; 1way ANOVA, Tukey´s multiple comparisons test (**a**) and unpaired t-test (**b**), * *p* < 0.05, **p < 0.01, ***p < 0.001

After 12 weeks of diabetes, the rats showed increased numbers of acellular capillaries compared to controls (NC 40.17 ± 5.85 AC/mm^2^ retinal area vs. DC 88.50 ± 11.74 AC/mm^2^ retinal area, *n* = 6), which was significantly reduced upon lixisenatide treatment (DC 88.50 ± 11.74 AC/mm^2^ retinal area vs. DC + Lixi 64.67 ± 11.83 AC/mm^2^ retinal area, *n* = 6) (Fig. 2a). Furthermore, pericyte numbers were reduced by 13% in diabetic rat retinae compared to non-diabetic controls (NC 2168 ± 219.4 PC/mm^2^ retinal area vs. DC 1885 ± 181 PC/mm^2^ retinal area, *n* = 6). Lixisenatide treatment lead to a protection from pericyte loss, as DC + Lixi animals had pericyte numbers similar to non-diabetic controls (NC 2168 ± 219.4 PC/mm^2^ retinal area vs. DC + Lixi 2120 ± 98.19 PC/mm^2^ retinal area, *n* = 6) (Fig. 2b).

**Fig. 3:**
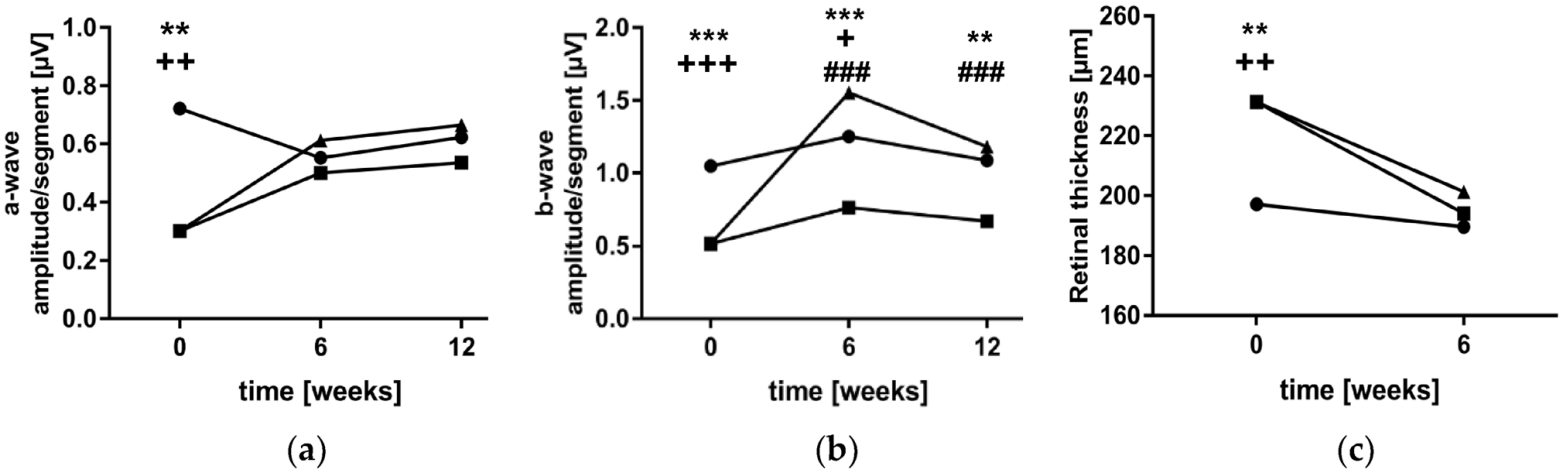
Assessment of neuroretinal function of lixisenatide. Multifocal electroretinography measurements of **a** a-wave amplitude (photoreceptor function) and **b** b-wave amplitude (inner retinal neurons) at 12 weeks. **c** OCT of retinal thickness at 6 weeks. ● NC = non-diabetic; ■ DC = diabetic; ▲ DC + Lixi = Lixisenatide-treated diabetic. Data are expressed as mean ± SD, 2way ANOVA, Tukey’s multiple comparisons test, *p < 0.05, **p < 0.01, ***p < 0.001. *NC versus DC, + NC versus DC + Lixi, # DC versus DC + Lixi

We assessed the neuroretinal function using mfERG. Photoreceptor function (a-wave amplitude) was unaffected by diabetes and unchanged upon lixisenatide treatment (DC 0.5360 ± 0.2044 a-wave amplitude/segment [µV] vs. DC + Lixi 0.6648 ± 0.1499 a-wave amplitude/segment [µV], *n* = 4) (Fig. 3a). After 12 weeks, lixisenatide treatment preserved the function of inner retinal neurons (b-wave amplitude) compared to untreated diabetic animals (DC 0.6683 ± 0.0824 b-wave amplitude/segment [µV] vs. DC + Lixi 1.1810 ± 0.1076 b-wave amplitude/segment [µV], *n* = 4–5) (Fig. 3b).

Retinal thickness was measured using OCT as an indicator of neurodegeneration. In diabetic animals, we observed an initial increased retinal thickness compared to non-diabetic animals due to osmotic effect or direct STZ treatment (NC 197.0 ± 11.53 retinal thickness [µm], DC 231.2 ± 14.65 retinal thickness [µm], *n* = 3–5). No structural changes were detected after 6 weeks in diabetic animals and in lixisenatide treated compared to diabetic animals (NC 189.5 ± 10.66 retinal thickness [µm], DC 194.0 ± 6.38 retinal thickness [µm] and DC + Lixi 201.2 ± 4.38 retinal thickness [µm], *n* = 4–5) (Fig. 3c).

### Macro- and microglial activation is mitigated by lixisenatide

**Fig. 4:**
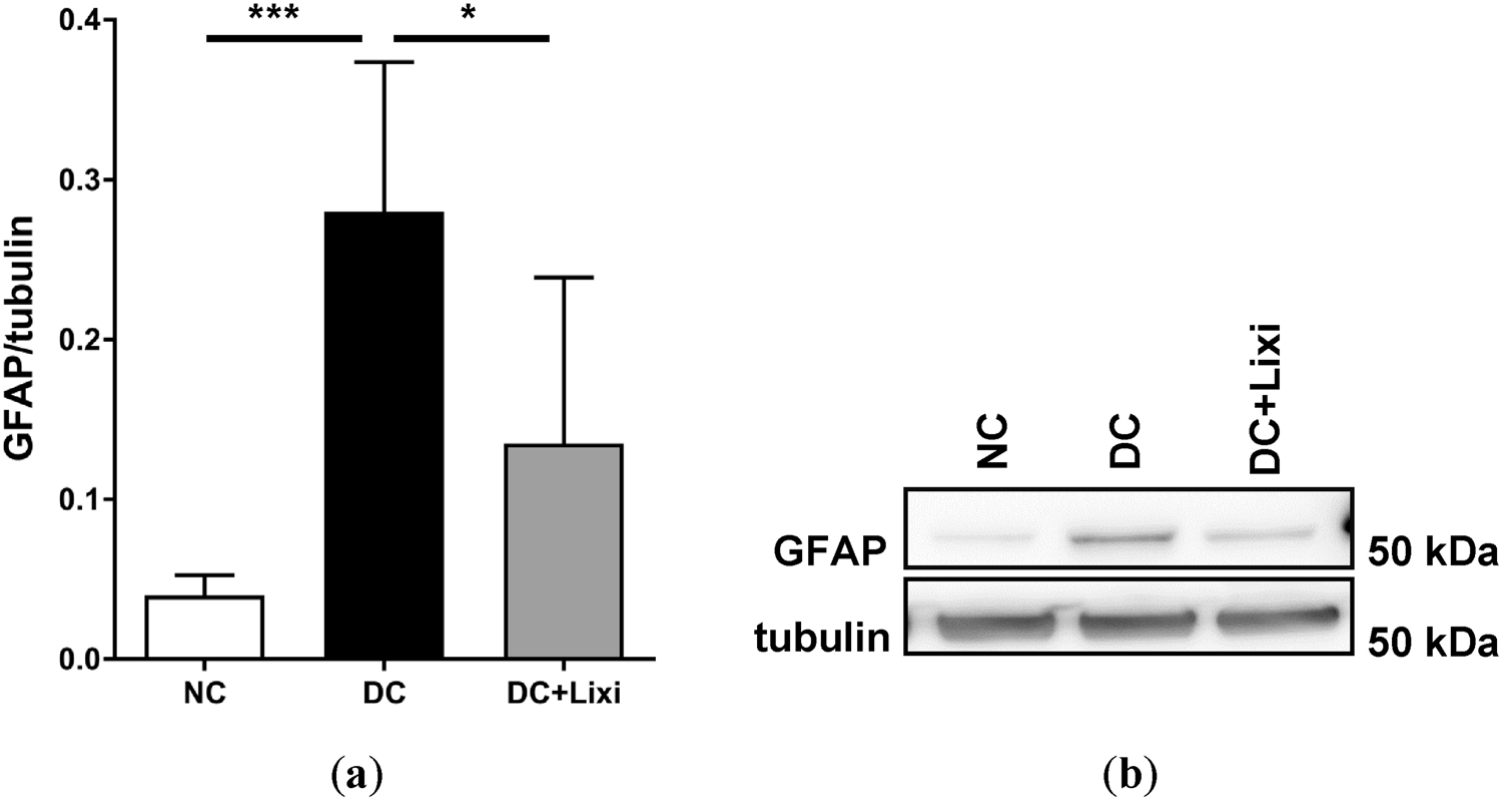
Quantification of glial activation. **a** Glial activation was quantified by Western blotting using GFAP (glial fibrillary acidic protein) as a marker (*n* = 5–6). **b** Representative blot for GFAP and *β*-Tubulin as loading control. NC = non-diabetic; DC = diabetic; DC + Lixi = Lixisenatide-treated diabetic. Data are expressed as mean ± SD, 1way ANOVA, Tukey´s multiple comparisons test, *p < 0.05, ***p < 0.001

To study the effect of lixisenatide on diabetic gliosis for macroglia activation (Müller glia), GFAP was used as a marker and quantified by Western blotting (Fig. 4a). In diabetic rats, the levels of retinal GFAP were 600% higher than in control animals, indicating Müller cell gliosis (NC 0.04 ± 0.01 vs. DC 0.28 ± 0.09, *n* = 5–6). This increased GFAP expression was reduced by 50% upon lixisenatide treatment in diabetic animals (DC 0.28 ± 0.09 vs. DC + Lixi 0.14 ± 0.10, *n* = 5–6) (Fig. 4b).

**Fig. 5:**
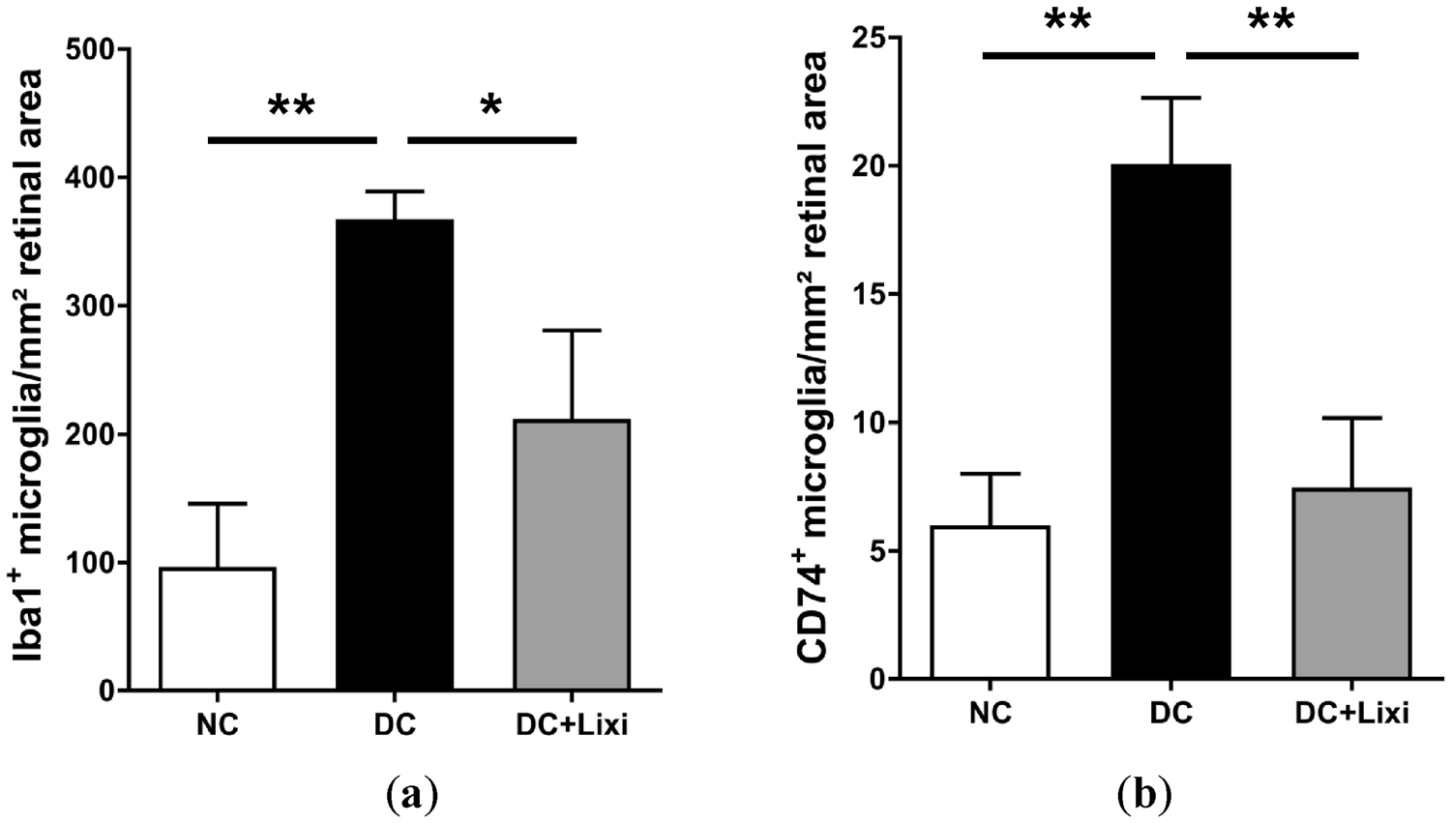
Quantification of microglial activation. Assessment of microglial activation by quantification of **a** Iba-1 + and **b** CD74 + positive cells (*n* = 3). NC = non-diabetic; DC = diabetic; DC + Lixi = Lixisenatide-treated diabetic. Data are expressed as mean ± SD, 1way ANOVA, Tukey´s multiple comparisons test, *p < 0.05, **p < 0.01

We assessed proliferation and activation of retinal microglia using immunofluorecense stainings for Iba1 + and CD74 + in retinal whole mount preparations. In diabetic animals, the number of microglial cells was significantly increased (NC 96.43 ± 49.44 microglia/mm^2^ vs. DC 367.2 ± 22.06 microglia/mm^2^, *n* = 3). Systemic treatment with lixisenatide reduced the number of microglia (DC + Lixi 211.7 ± 69.0 microglia/mm^2^, *n* = 3) (Fig. 5a). In line with the proliferation of microglia in retinae of diabetic animals, the numbers of CD74 + positive activated microglia were significantly higher in diabetic rats compared to non-diabetic controls (NC 5.996 ± 2.022 CD74 + cells/mm^2^ vs. DC 20.07 ± 2.579 CD74 + cells/mm^2^, *n* = 3). Lixisenatide treatment completely prevented the increase in microglial activation in diabetic animals (DC + Lixi 7.46 + 5 ± 2.706 CD74 + cells/mm^2^, *n* = 3) (Fig. 5b).

### Lixisenatide treatment normalizes gene expression changes in diabetic animals

**Fig. 6:**
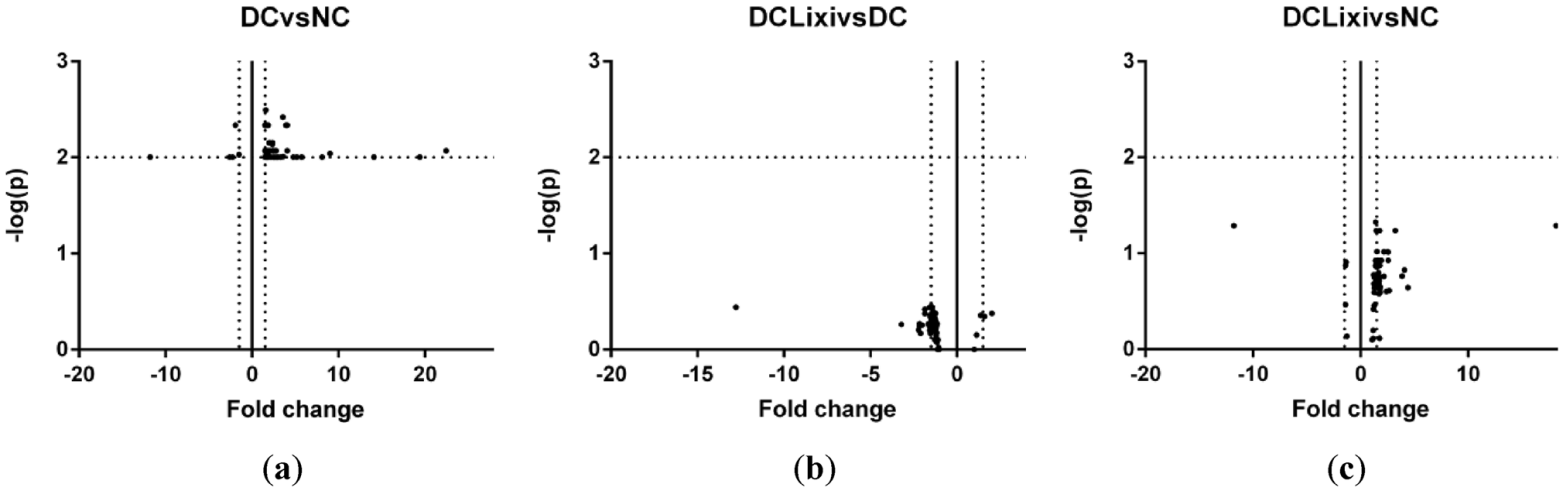
Volcanoplot of genes detected by RNAseq of retinae (*n* = 6). **a** 66 genes show significant levels of regulation between the untreated diabetic animals and the healthy control animals (DC vs. NC). **b** Lixisenatide normalizes diabetic gene regulation. **c** The differences between untreated and Lixisenatide-treated diabetic animals are not statistically significant (DC + Lixi vs. DC)

To identify gene regulatory mechanisms mediating the protective effects of lixisenatide, we performed RNA sequencing of rat retinae (*n* = 6). Diabetes induced gene expression change in a total of 66 genes. Lixisenatide normalized the expression of these genes in diabetic animals (DC + Lixi vs. NC) to the level of healthy control animals. The effect of treatment on diabetic animals was moderately regulated (Fig. 6a–c).

A representative selection of the genes shown in Fig. 6 is summarized in Table 1.

**Table 1:** Genes with expression changes

Gen-Symbol	Gen-name	Fold change		
		DC versus NC	DC + Lixi versus NC	DC + Lixi versus DC
Gfap	Glial fibrillary acidic protein	14.10	4.39	− 3.21
Cebpd	CCAAT/enhancer binding protein delta	9.02	4.07	− 2.22
Chi3l1	Chitinase 3 like 1	5.17	2.39	− 2.16
A2m	Alpha-2-macroglobulin	4.76	2.36	− 2.02
Clec3b	C-type lectin domain family 3, member B	4.06	2.57	− 1.58
Pgf	Placental growth factor	3.93	2.50	− 1.57
Mt2A	Metallothionein 2A	3.60	3.21	− 1.12
Fos	FBJ osteosarcoma oncogene	3.32	2.17	− 1.53
Tnfrsf1a	TNF receptor superfamily member 1A	2.95	1.82	− 1.62
Cd6	Cd6 molecule	2.69	1.72	− 1.57
Ednrb	Endothelin receptor type B	2.48	1.77	− 1.40
Mlc1	Megalencephalic leukoencephalopathy with subcortical cysts 1	2.46	1.75	− 1.40
Hcls1	Hematopoietic cell specific Lyn substrate 1	2.16	1.86	− 1.16
Ets2	ETS proto-oncogene 2, transcription factor	2.14	1.58	− 1.35
Tshr	Thyroid stimulating hormone receptor	1.96	1.55	− 1.27
Pim1	Pim-1 proto-oncogene, serine/threonine kinase	1.93	1.63	− 1.19
Aqp4	Aquaporin 4	1.71	1.47	− 1.16
Rras	Related RAS viral (r-ras) oncogene homolog	1.70	1.39	− 1.22
Slc1a3	Solute carrier family 1 member 3	1.65	1.31	− 1.26
Msn	Moesin	1.64	1.35	− 1.22
Bend4	BEN domain containing 4	1.61	1.20	− 1.34
Plat	Plasminogen activator, tissue type	1.59	1.39	− 1.14
Ltbr	Lymphotoxin beta receptor	1.56	1.28	− 1.22
Ier3	Immediate early response 3	1.54	1.46	− 1.06
Adamtsl1	ADAMTS-like 1	1.50	1.21	− 1.24

### Ets2 is identified as a regulator of inflammatory genes

**Fig. 7:**
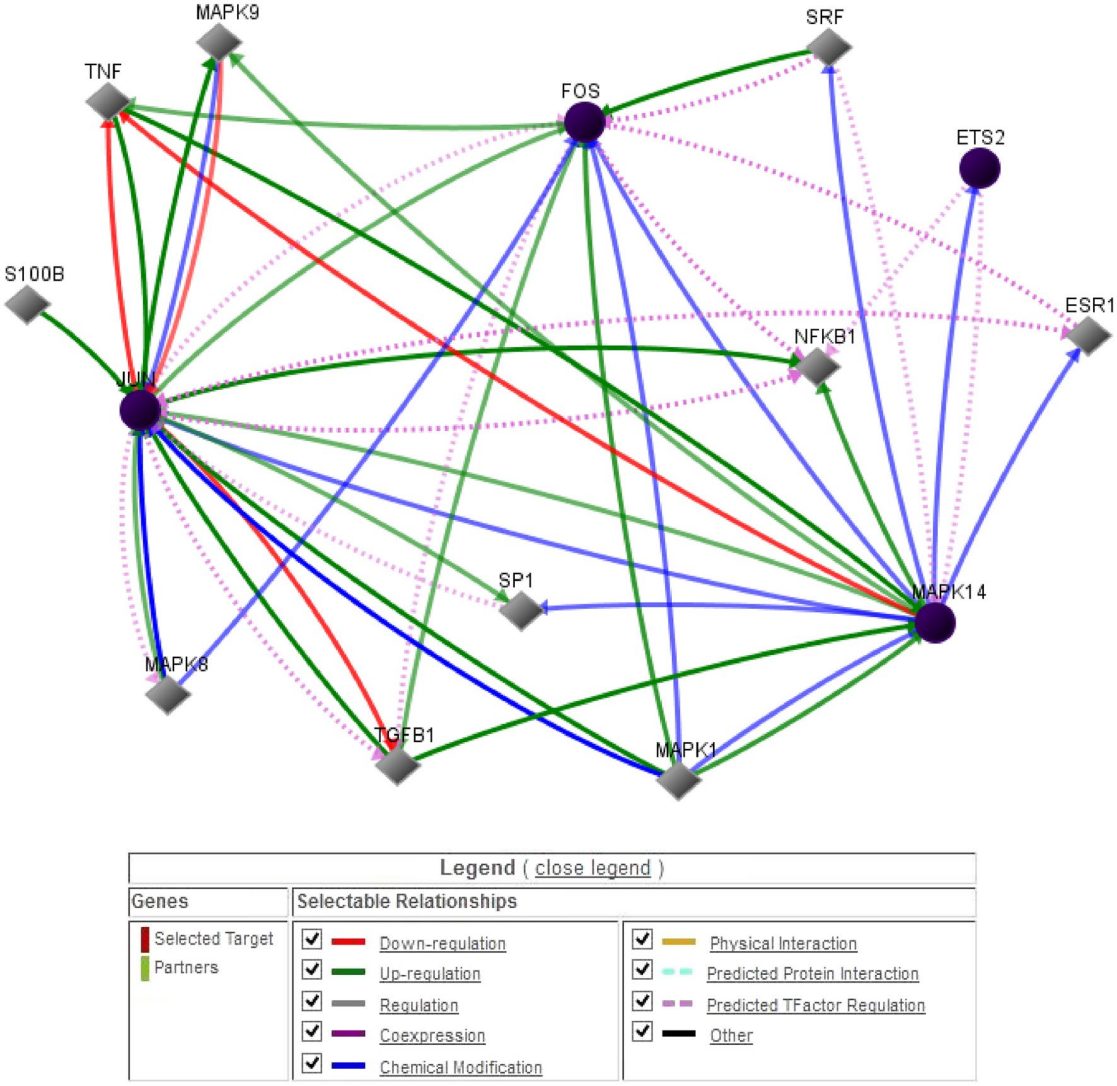
Regulatory network of astrocyte-annotated genes. The genes Ets2, FOS and JUN are phosphorylated via p38/MAPK14 (blue arrows). MAPK14 and JUN lead to the upregulation of NFKB1 (green arrows) (Color figure online)

To identify the annotations and the nature of the relationships involved in the genes, in silico analyses were performed using GNCPro. Ets2 was identified as a regulator of several inflammatory genes, predicting interactions by co-expression, regulation, physical interaction, predicted protein interaction or predicted transcription factor regulation. The data obtained from retina RNA sequencing were specified to retinal cell types using in silico cell-type analysis of the identified genes. Here, the identified genes were annotated to astrocytes (76%), microglia (45%), endothelial cells (42%) and neurons (26%). After identifying astrocytes as the primary target structure from the in silico analysis, a regulatory network for astrocyte-annotated genes was performed. This predicted a relationship between the genes Ets2, FOS and JUN, which are involved in the regulation of NFKB1 (Fig. 7).

### Lixisenatide-reduced ROS formation under HG condition in *C. elegans* in a concentration-independent manner

**Fig. 8:**
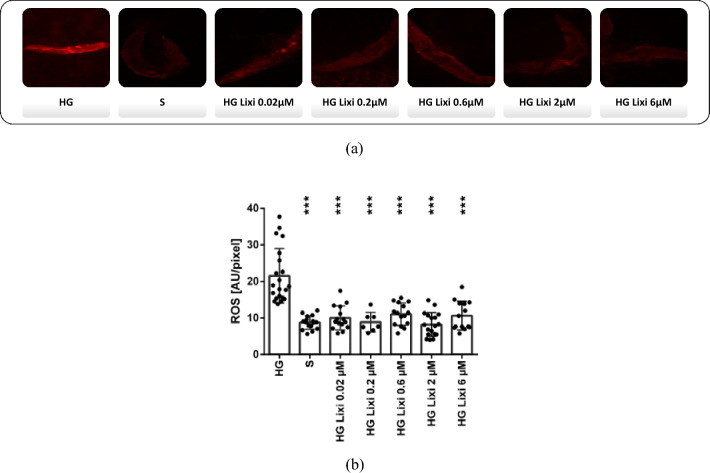
Formation of reactive oxygen species (ROS) in *C. elegans*. Nematodes were cultured under standard (S) or high glucose (HG) conditions. For additional treatment, lixisenatide was added at concentration of 6 µM, 2 µM, 0.6 µM, 0.2 µM and 0.02 µM to the high glucose condition (HG + Lixi). ROS formation was detected by confocal laser scanning microscopy (CLSM) of ethidium-labeled nematodes at 12 days of age and quantified by calculation of mean pixel values [arbitrary units (AU)/pixel]. Shown are the results of one representative experiment from three independent experiments. **a** Representative CLMS images of the respective groups. **b** Summary of quantified ROS formation. Shown are MW ± SD (*n* = 7–21). Significance against HG condition was calculated using Tukey's multiple comparisons test, ***p < 0.001

The short lifespan, an accessible and well-characterized nervous system, the absence of a vascular system and the high homology to human genes (40%) make *C.* *elegans* as a model organism very attractive [25, 26]. The absence of a vasculature and the fact that neurons constitute over one third of its total somatic cell population allows it to serve as a model for the study of neurodegenerative processes [27], such as a surrogate for the isolated study of neuronal function in the pathogenesis of DR. *C.* *elegans* also lacks a GLP-1R orthologue, so the immediate properties of GLP-1 RAs, such as lixisenatide, can be analyzed from its GLP-1R-mediated effects. Cultivation of nematodes under high glucose (HG) condition causes increased ROS formation compared to standard (S) condition (S 8.79 ± 1.84 AU/pixel, HG 21.54 ± 7.46 AU/pixel, *p* < 0.001), which was significantly reduced upon Lixisenatide treatment independent of concentration (HG Lixi 0.02 µM 10.03 ± 3.27 AU/pixel; HG Lixi 0.2 µM 8.87 ± 2.70 AU/pixel; HG Lixi 0.6 µM 11.05 ± 3.06 AU/pixel; HG Lixi 2 µM 8.26 ± 3.26 AU/pixel; HG Lixi 6 µM 10.66 ± 3.95 AU/pixel). Lixisenatide treatment under HG conditions reduced ROS formation to normal levels (Fig. 8 a-b).

## Discussion

Diabetic retinopathy is a disease of the entire retinal neurovascular unit (NVU). The cause of the loss of pericytes and endothelial cells is a combination of reactive metabolites through chronic hyperglycemia, mitochondrial overproduction of ROS and AGEs [28, 29]. Inflammatory and proapoptotic signaling pathways are also involved as a cause of loss [28]. Several mechanisms have been suggested as responsible for glucose-mediated damage. Each of these mechanisms has been studied independently of the others. The Unifying Hypothesis made clear that all these seemingly unrelated mechanisms are due to a single process triggered by hyperglycaemia: the overproduction of reactive oxygen species by the mitochondrial electron transport chain [30].

In this study, it was shown that lixisenatide has a protective effect on the retina (reduction of acellular capillaries) but does not reduce the AGE precursors MG level, while in *C. elegans* an antioxidant effect was measured. The discovery that lixisenatide has protective properties on the entire neurovascular unit even though methylglyoxal levels in the retinas remain high has not been observed previously and is novel. Independent of metabolic effects, lixisenatide was protective on retinal vasoregression, neuronal dysfunction and glial activation.

Current treatments for retinopathy are not fully effective in delaying or stopping the development of disease. Laser therapy and intraocular injections of anti-VEGF agents are the established treatments, which are normally only used in advanced disease stages when microvascular abnormalities are already observed. However, these treatments are invasive and can have significant side effects [31]. Accordingly, a better understanding of the molecular mechanisms underlying the pathogenesis of retinopathy is essential. There is a dozen of classes of drugs approved for the treatment of T2D, including insulin, DPP4 inhibitors and GLP-1 RA. A significant number of potential therapeutic targets for the treatment of diabetic complications have been proposed using current rodent model, but the encouraging results obtained in preclinical studies have failed thus far in clinical trials [32]. The UK Prospective Diabetes Study (UKPDS) has demonstrated the close relationship between blood glucose levels and the development and progression of DR. The clinical study analyzed patients with type 2 diabetes mellitus (T2DM) between 1977 and 1997. It was shown that intensive blood glucose control reduced DR by 25% compared to conventional treatment. An intensive insulin therapy slowed progression of diabetic retinopathy by 54% [33]. Dietrich et al. [17] performed a study in STZ diabetic Wistar rats with linagliptin (DPP4 inhibitor) on the neurovascular unit of the retina and demonstrated the protective effect on the microvasculature of the diabetic retina, due to most probably a combination of neuroprotective and antioxidant effects on the neurovascular unit. The DDP4 inhibitor in this study had the potential to reduce vascular protection by inhibiting GLP-1 degradation products such as GLP-1(9–37) and GLP-1(9–36) amide, as the degradation products of active GLP-1 are supposed inhibitors of mitochondrial ROS overproduction [34, 35].

In contrast to the study using the DPP-4 inhibitor linagliptin, methylglyoxal levels in the rat retinae remained unaffected and the antioxidant properties of the GLP-1 RA lixisenatide and the positive effect on neuronal functions in *C. elegans* was demonstrated [17]. *C. elegans* which lacks a GLP-1 receptor serves as a surrogate for demonstrating antioxidant properties of lixisenatide independently of GLP receptor functions.

The expression of a functional GLP-1 receptor in the retina, however, is assessed differently. While there is some published evidence for a functional receptor in the mouse [36, 37], data have been published that using specific molecular tools only few ganglion cells in the healthy human retina express the receptor, while in advanced diabetic retinopathy, the receptor is no longer detectable [29, 38, 39]. We therefore suggest that in synopsis of the available data, we consider the observed effects of lixisenatide to be largely independent of a potentially existing GLP-1 receptor.

Oxidative stress plays a central role in the pathogenesis and progression of diabetic retinopathy. During the process of NVU impairment in diabetic retinopathy, reactive oxygen species (ROS) are generated due to the imbalance between free radical formation and degradation. Most pathological pathways, e.g., the polyol pathway, the hexosamine pathway, the AGE pathway and the PKC pathway, can result in oxidative stress [28]. In addition, there are synergistic effects of all the above changes, leading to extensive retinal cell damage and thus progression of DR [40]. Several studies have investigated the effects of antioxidant supplementation on diabetes in general and on its complications. Extensive reviews [41–46] have evaluated the finding:

Antioxidants appear to be promising in inhibiting the development of DR in animal models. For example, thiamine (vitamin B1) is an essential cofactor for several enzymes in the Krebs cycle and a protective substance against metabolic damage caused by diabetes. Previous studies have shown that thiamine and benfotiamine (a lipophilic form of thiamine) prevent retinal pericytes from apoptosis induced by mitochondrial dysfunction, as well as retinal histopathology and other metabolic pathways associated with the development of diabetic retinopathy[47, 48]. There are other known antioxidants that are thought to have a high therapeutic potential in diabetic retinopathy. To name a few other examples Aminoguanidine (Inhibits the accelerated death of retinal capillary cells and development of retinopathy, inhibits lipid peroxidation and AGEs formation); Lipoic acid (Attenuates the apoptosis of retinal capillary cells and acellular capillaries, decreases the levels of nitrotyrosine, VEGF and oxidatively modified proteins, activation of NF-kB) and Vitamin C/E (Reduce neovascularization, prevent the inhibition of retinal GR, GPx and SOD activities) have also shown positive properties on the DR [46]

As lixisenatide likewise inhibits inflammatory and proapoptotic signal pathways suggesting that oxidative Fstress induces a cascade in which the latter induces the former. Both macroglial and microglial activation were reduced by lixisenatide. It is already known that activated microglia produce glutamate, iNOS (Inducible Nitric Oxide Synthase) and pro-inflammatory cytokines such as IL-1β, IL-6, IL-12 and TNFα, which can result in the activation of caspases, accelerating cell retinal death [49]. Using RNA sequencing, 66 genes were identified that showed significant gene expression changes in diabetic animals compared to healthy controls. Lixisenatide resulted in partial inhibition of several gene activations and normalized the expression of these genes to that of the healthy control animals. In general, the database (RefSeq status at the National Center for Biotechnology Information (NCBI): http://www.ncbi.nlm.nih.gov/refseq/) of the function of the affected genes indicated that some of these genes play a role in immune and inflammatory responses such as *Cebpd, Chi3l1, A2m, Tnfrsf1a, Cd6*, participate in the regulation of cell proliferation, differentiation, transformation and apoptosis (*Mt2A, Fos, Tnfrsf1a, Pim1, Ltbr, Ier3*). Some regulated genes encode growth factors (*Pgf, *Ets2), membrane proteins (*Tshr, Aqp4, Msn*) and proteins which can act as antioxidants (*Mt2A*). The protective gene *Mt2A* was moderate downregulated and the above-mentioned harmful genes were downregulated by lixisenatide treatment. In addition to the gene for astrocyte markers (*GFAP*), a gene (*Rras*) was identified that encodes a GTPase involved in various processes such as angiogenesis, vascular homeostasis and regeneration, cell adhesion and neuronal axon guidance. Furthermore, some genes encode glutamate transporter (*Slc1a3*), G protein-coupled receptor whose ligand is highly vasoactive, and an enzyme (*Plat*) that play a role in cell migration and tissue remodeling. The observed changes in gene expression between the lixisenatide-treated and diabetic groups were not statistically significant. Nevertheless, lower gene expression of harmful genes was observed in the lixisenatide-treated group. This suggests that the benefit of lixisenatide is not in the regulation of single gene but is rather a cluster effect in the regulation of several genes.

Using in silico analyses, a clustering was detected for the regulated genes. Here, Ets2 was identified as a regulator of some of these genes. Ets2 encodes a transcription factor which regulates genes involved in development and apoptosis. The encoded protein is also a prooncogene and has been shown to be involved in the regulation of telomerase. A pseudogene of this gene is located on the X chromosome. Alternative splicing leads to several transcript variants (RefSeq database: http://www.ncbi.nlm.nih.gov/refseq/; Jan 2012). In diabetic retinopathy, this may indicate that Ets2 is involved in pathological processes.

Cheng et al. explored the role of Ets2 in the mouse model of vulnerable plaque and in endothelial cells. Ets2 expression was increased in the mouse model under atherogenic conditions. Overexpression of Ets2 promoted lesion growth with formation of neovascularization, haemorrhage and destabilization of plaques. In contrast, knockdown of Ets2 using a lentiviral shRNA construct promoted lesion stabilization. In endothelial cell cultures, Ets2 expression and activation responded to the cytokine TNF*α*. The in vitro study showed that Ets2 was critical for TNF*α*-induced expression of monocyte chemoattractant protein 1, interleukin-6 and vascular cell adhesion molecule 1. Ets2 also promoted microvessel formation and enhanced TNF*α*-induced loss of vascular endothelial integrity. Evaluation of a mouse retinal model confirmed the role of Ets2 in regulating vascular inflammation and endothelial leakage [50].

The study by Chung et al*.* [51] investigated the potential anti-inflammatory effects of lixisenatide on the retina in an animal model of early type 2 diabetes. The results showed that lixisenatide treatment reduced inflammation in retinas of diabetic mice, independent of hyperglycemia. The authors concluded that lixisenatide may have potential as a therapeutic agent for reducing inflammation in the early stages of diabetic retinopathy, which could potentially prevent or delay the development of more severe forms of the disease.

Lixisenatide treatment decreased Ets2 expression in diabetic animals which may have led to protection of the NVU by inhibition of vascular inflammation (inflammatory cytokines) and endothelial damage in diabetic rat retinae.

An in silico cell-type analysis of the identified genes showed that the majority was annotated to astrocytes (76%). In the retina, astrocytes, Müller cells, microglia, and pericytes normally inhibit endothelial cell proliferation and maintain vascular stability and the inner blood–retinal barrier [52]. The proper functioning of all elements of this unit is essential for the normal function of the retina, as it enables the neuronal retina to adapt to different physiological conditions. Glial cells are the interface between the vascular system and the neurons [3]. Astrocytes have multiple functions and are a key connector between blood capillaries and neurons. They regulate the health of neurons by controlling neurotransmitters such as glutamate or adenosine. Astrocytes are not only in contact with neurons, but also with blood vessels. They are considered to control local blood flow to supply nutrients to the neurons and regulate synaptic activities. Astrocytes also contribute physically and functionally to the blood-retina barrier and its permeability to factors such as the molecular trafficking of glucose or proteins. Therefore, astrocytes are central to the function of a healthy functioning NVU [53, 54]. This is no longer the case, for example under chronic hyperglycemia and the NVU is impaired [3]. Astrocytes induce inflammation during the development of DR by upregulating the expression of cytokines and chemokines which may lead to degradation of blood-retina barrier. Due to diabetes, the dysregulation of metabolic pathways leads to an accumulation of ROS, which in turn causes astrocyte activation. In turn, this is reflected by increased proliferation, migration, GFAP expression and secretion of proinflammatory factors, induced by the activation of TNF-*α* and the Nuclear Factor Kappa-B (*NFkB*) pathway, as well as an increase in oxidative stress [55]. The activated *NFkB* further binds to the nuclear DNA and overexpresses various genes, leading to the production of free radicals, cytokines, pro-apoptic and pro-inflammatory molecules. All these factors can lead to apoptosis, neuronal degeneration, endothelial dysfunction, inflammation and circulatory disturbances and finally to diabetic retinopathy [56].

For these astrocyte-identified genes, a regulatory network was created and a relationship between the genes Ets2, *FOS* and *JUN* were predicted, which is involved in the regulation of *NFKB1*.

Besides the isolated evaluation of GLP-1R independent antioxidative effects of lixisenatide, under hyperglycemic condition, the reduction of AGE accumulation in *C.* *elegans* was observed (data not shown). This observation is also consistent with the literature and suggests the direct antioxidant effect and indirect AGE effect of lixisenatide on oxidative stress by slowing down the Maillard reaction. The accumulation of ROS leads to inflammation, impairment of NVU and cell death. The changes in various transcription factor signals, such as *NFKB*, are induced by oxidative stress [57].

**Fig. 9:**
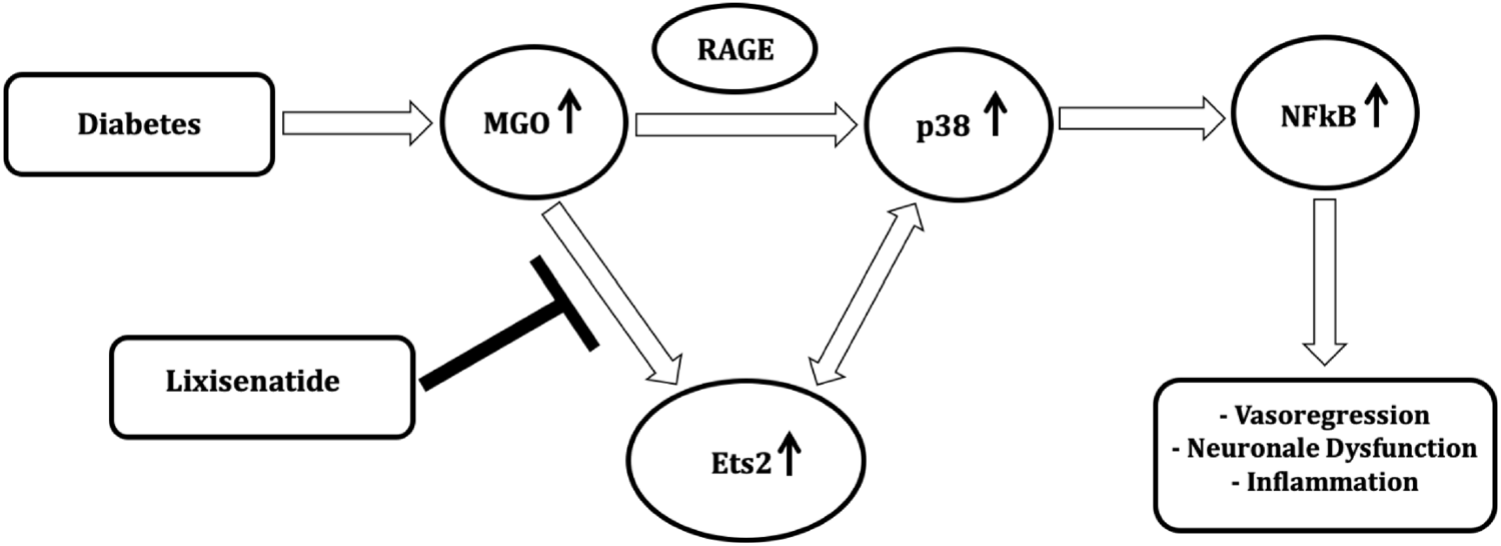
Potential mechanism of lixisenatide action. In diabetes, there is an accumulation of MG/AGE, which activates p38/MAPK14 via the RAGE pathway and leads to an increase in NFkB levels. This leads to vasoregression, neuronal dysfunction and inflammation. Since the amount of retinal MG was not affected by lixisenatide, the protective effect of lixisenatide is probably mediated downstream from the MG/AGE-RAGE. Lixisenatide inhibits the expression of Ets2 and thus can inhibit the activation of p38/MAPK14

In diabetes, there is an activation of p38/MAPK14 via the RAGE pathway and can lead to an increase in *NFKB* levels. This, in turn can induce vasoregression, neuronal dysfunction and inflammation [28]. Since MG was not affected by lixisenatide, the protective effect of lixisenatide may be mediated downstream of the MG-induced RAGE pathway. Lixisenatide inhibits the expression of Ets2 and thus may bypass the RAGE pathway and directly activate of p38/MAPK14. Therefore, we propose the working model in Fig. 9.

The protective effect of lixisenatide is probably not mediated by influencing a single pathway, but via a slight cluster effect that accumulates in its protective effect. The potential inhibition of Ets2 upregulation and the associated reduction of Ets2-induced gene activation of various cell-type-specific genes probably reduce, for example, the activation of *NFkB* and the p38 MAPK.

Overall, the results in this study suggest that lixisenatide is potentially beneficial for the retinal neurovascular unit in diabetes. Although the experimental results achieved are not completely transferable to humans ("lost in translation"), some aspects are transferable and these are of immense importance for patients, as there is a need for new therapeutic options that are able to prevent or slow down the development and progression of diabetic retinopathy. Lixisenatide may have potential as a therapeutic agent for reducing oxidative stress and inflammation (GLP-1 receptor independent) in the early stages of diabetic retinopathy, which could potentially prevent or delay the development of more severe forms of the disease.

## Conclusions

Our data suggest that the GLP-1 receptor agonist lixisenatide has a protective effect on the microvasculature of the diabetic retina, most likely due to a combination of neuroprotective and antioxidative effects of lixisenatide on the neurovascular unit.

## Data Availability

The RNA Seq data discussed in this publication have been deposited in NCBI's Gene Expression Omnibus [58] and are accessible through GEO Series accession number GSE236333 (https://www.ncbi.nlm.nih.gov/geo/query/acc.cgi?acc=GSE236333).
